# Prenatal MRI visualisation of the aortic arch and fetal vasculature using motion-corrected slice-to-volume reconstruction

**DOI:** 10.1186/1532-429X-18-S1-P180

**Published:** 2016-01-27

**Authors:** David Lloyd, Bernhard Kainz, Joshua F van Amerom, Maelene Lohezic, Kuberan Pushparajah, John M Simpson, Christina Malamateniou, Joseph V Hajnal, Mary Rutherford, Reza Razavi

**Affiliations:** 1 Evelina Children's Hospital, London, United Kingdom; 2 King's College London, London, United Kingdom

## Background

The antenatal diagnosis of vascular abnormalities such as coarctation of the aorta may allow for more timely provision of what can be life-saving postnatal care. Fetal MRI offers the potential to compliment conventional antenatal assessment of the extracardiac vasculature, which can be difficult to assess by ultrasound.

## Methods

Using overlapping multi-slice 2D single-shot fast spin echo sequences (Philips, 1.5T, TR = 15000 ms, TE = 100 ms, flip angle = 90 degrees, voxel size = 350 x 350 mm, slice thickness = 2.5 mm, SENSE factor = 2, partial Fourier-factor 5/8, slice duration 468 ms) in a fetus at 36 weeks gestation, we applied a novel GPU accelerated super-resolution algorithm for slice-volume registration to the oversampled data to compensate for fetal movements between slices.

## Results

A 3D dataset was generated with an isotropic voxel size of 0.4 mm for visualisation of fetal structures. Using this we were able to clearly show the relationship of the great vessels, demonstrating hypoplasia of the aortic arch and a high risk of coarctation. Following delivery, critical coarctation of the aorta was confirmed. The baby went on to have surgical repair in the neonatal period.

## Conclusions

Uncontrolled fetal movement represents a major challenge to more widespread adoption of fetal cardiovascular MRI. Automated motion-corrected 3D volume reconstructions could greatly increase the diagnostic utility of antenatal MRI in the future.

**Figure 1:**
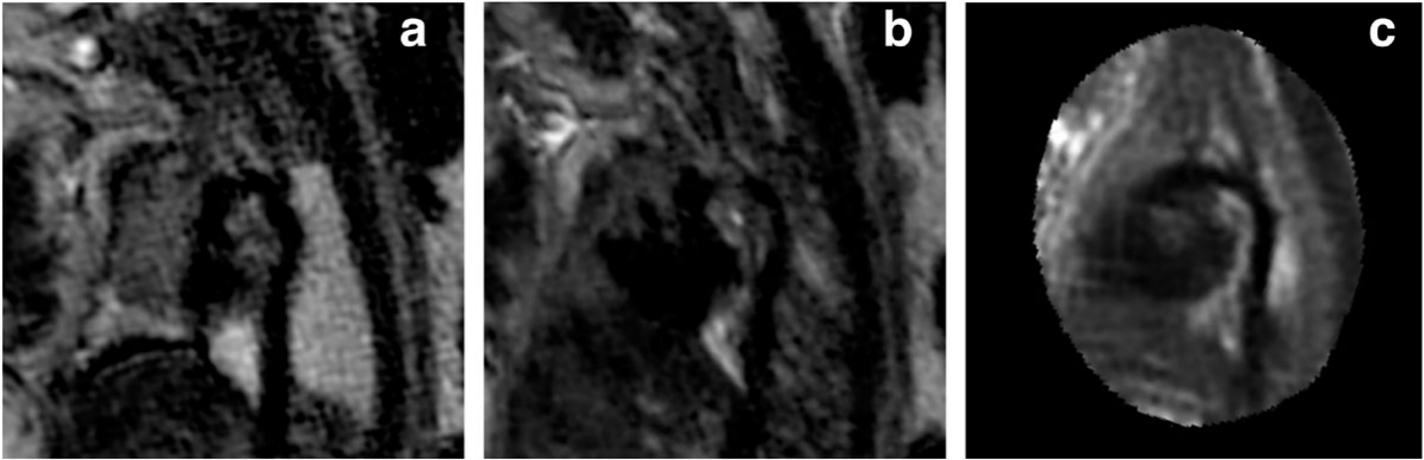
**The aortic and ductal arches in a 36 weeks fetus as visualised from a.) a single-shot fast spin echo MRI image; b.) a (motion-corrupted) 3D volume derived from the concomitant multi-slice stack; and c.) the resultant 3D volume after application of a GPU accelerated super-resolution algorithm for slice-volume registration (using multiple stacks)**. In the final image, the hypoplastic aortic arch is now clearly visualised superior to the dominant ductal arch.

